# “Lead like a woman”: strengthening healthcare, medical imaging, and oncology through female leadership

**DOI:** 10.1093/bjr/tqaf310

**Published:** 2026-01-12

**Authors:** Christina Malamateniou, Patrizia Cornacchione

**Affiliations:** CRRAG Research Group, School of Health and Medical Sciences, City St George’s University of London, London ECV1 0HB, United Kingdom; European Federation of Radiographer Societies, Cumieira 5030-058, Portugal; European Society of Medical Imaging Informatics, Vienna 1010, Austria; Department of Neuroimaging, King’s College London, London SE59RS, United Kingdom; Dipartimento di Diagnostica per Immagini e Radioterapia Oncologica, Fondazione Policlinico Universitario Agostino Gemelli IRCCS, Rome 00168, Italy; Dipartimento di Scienze Radiologiche ed Ematologiche, Università Cattolica del Sacro Cuore, Rome 00168, Italy

**Keywords:** leadership, women, medical imaging, oncology, diversity, inclusion, collaboration, integrity, sustainability

## Abstract

Women are overall underrepresented in high-level leadership positions across organizations, particularly in the fields of healthcare, medical imaging, and oncology, despite the majority of the workforce in these industries being female. Women leaders often remain in these roles for shorter times, despite evidence documenting their value in increasing team wellbeing, productivity and collaboration and supporting organizational integrity, sustainability, diversity and inclusion. The reasons are complex, but often due to a lack of ongoing support or poor local culture. There is an urgent need to harness the potential of female leadership, not only to leverage equity and diversity, but mainly to solve the complex healthcare challenges of our diverse society now and in the future. Customized training and mentoring, clearer career pathways, flexible work, workplace adaptations and male allyship are key to raising, nurturing and supporting female leaders in the long term.

## Introduction and rationale

Despite women making up half of the world’s population, their presence in leadership positions in different sectors is largely under-represented.^1^ There are known challenges, gaps, barriers, conscious and unconscious bias, and implicit or explicit cultural norms that are responsible for this.^2^ The majority of academic and wider literature is rife with papers that recount these gaps and challenges and propose ways to support women entering or maintaining a leadership position.^1–5^ There is an increase in evidence-base that celebrates the attributes, characteristics, and positive impact of women leaders in society, in general and healthcare, in particular. However, many of these successes remain anecdotal and unpublished. The aim of this commentary is to contextualize the current status of women’s leadership, and celebrate the positive attributes and impact that women leaders bring to society. There will be a focus on healthcare, medical imaging and oncology, with suggestions to minimize the leadership gap and empower female leaders in the future for public and patient benefit.

## Gender gap in leadership

Although there has been some progress in minimizing leadership disparities in the last decade, gender imbalance on top managerial positions persists, particularly so in low- and middle-income countries.^6^ Women leaders continue to face unprecedented challenges to their authority and value, greater than their male counterparts while at the same time they are afforded less psychological safety. They also experience bias, that extends from gender to other intersectional areas of their identity, like race, sexual orientation, ethnicity, disability, neurodiversity and more. Women leaders, and more so those from different ethnic minorities, are less likely to report managerial support, or allyships by their wider team, that would otherwise help them deliver impact and reach their full potential.^6–8^ Women leaders are also less likely to report explicit mentorship or sponsorship by their seniors, which might include praise of their performance, wider advocacy within the team, moral support or discussions around salary equity.^9^^,^^10^ Compound influence of racial and gender biases hinders the advancement of minority female leadership by perpetuating stereotypical behavioural schemas, leading to persistent discriminatory outcomes.^11^ Recruitment, promotion, and retention processes for leadership positions are adversely affected, primarily due to bias, inadequate support mechanisms, and discrimination.^12^ Instead, female professionals report being subjected to more demeaning comments, verbal or sexual abuse, bullying, harassment, discrimination and microaggressions in the workplace.^6^^,^^13^^,^^14^

Only 10% of Fortune500 companies are led by women.^6^ In 2015, 26% of leadership positions within the finance sector were given to women, increasing to 32% in 2021. Across industries, in 2022, the number of women hired into director or higher roles reached 37%, an increase of 6% since 2015. Women are significantly underrepresented in the STEM industry, with only 29.2% of the workforce being made up of women.^15^ Despite making up more than 70% of the healthcare workforce and 90% of the nursing and midwifery workforce, they hold just 25% of leadership roles.^16^ Women also occupy less than 30% of medical director roles and make up just 22% of artificial intelligence professionals globally.^17^^,^^18^ They are also less likely to stay in their leadership position for longer and more likely to resign than a male counterpart. Even when they finally manage to ascend in a leadership role, despite all challenges and obstacles on the way, the lack of robust support sets them to fail more often than men.^3–5^^,^^19^

The gender gap in academia, particularly in scientific research and leadership, is a well-documented and persistent issue.^19–23^ Women in academic radiology and other scientific disciplines related to medical imaging and oncology (radiography, medical physics, biomedical engineering) continue to face disparities in authorship (first or last author), conference attendance, faculty rank, leadership opportunities, and compensation and their careers are the first ones to be impacted by large-scale crises, like the COVID-19 pandemic.^24–30^ In 2019, women marginally increased their representation at professorial level in the EU to 26% (from 24% in 2016) and the proportion of female heads of higher education institutions (HEIs) stood at 23.6% in 2019.^20^^,^^21^ Despite progress over the past decades, the pace of change remains slow, and structural barriers still prevent gender equity at the highest levels of academia.

Several theories have been explored to explain gender disparities, many of which revolve around the toll of women assuming caring commitments for family and elderly parents; however, the truth might be a bit more complex. Professor Alice Eagly, an eminent U.S. scholar, who spent her career understanding the root in gender discrepancies in leadership, has highlighted the *role congruity theory of prejudice*.^7^^,^^8^ This proposes that perceived incongruity between the female gender role and leadership roles leads to 2 forms of prejudice: (1) less favourable perceptions of women over men as potential occupants of leadership roles and (2) less favourable perceptions and behaviours for those already in leadership roles when they happen to be a woman. As a result, there are more negative attitudes towards female than male leaders and towards women as potential leaders, making it significantly more difficult for women to get to the top positions and to achieve success in leadership roles.^7^ Other studies have discussed that barriers across three broad domains: socially constructed *perceptions of capability, capacity, and credibility* of women prevent them from reaching the higher echelons of leadership.^31^

## The attributes of women leaders and their impact on society

Despite known barriers to accessibility for women, female leadership tends to add a unique signature of both communal traits, such as compassion, responsibility, inclusion, and improved key performance indicators in relation to personal and team effectiveness.^32^ Women are more likely to employ transformational leadership styles and to inspire people to work towards achieving the shared mission of a team or broader organization, compared with men.^6^^,^^14–16^^,^^33^ Literature supports that when women are leading, team collaboration is improved; this is mainly due to more democratic turn-taking in conversations, that enables equal contributions to discussions. Women also are better at harnessing collective intelligence, which is the group’s ability to work together to solve a common problem.^34^ Another study showed that women ranked better (or equal) to men in many leadership related traits, such as honesty, intelligence, compassion, expressiveness, communication, and creativity; they only seemed to lag in decisiveness.^35^ More recent work in 2018 demonstrated that women leaders are now increasingly perceived as equal or more competent than men compared to studies in the 50s and 60s, for instance.^36^ This related to different attributes, such as compassion, agency, and competence (ie, intelligence, creativity).^36^ Female leaders also increase expectations for fairer treatment among team members and are more likely to cultivate a climate of trust and mutual support.^37^

Compelling evidence from published studies and reports also show that when more women are empowered to lead, everyone benefits. Decades of studies show women leaders help increase productivity, enhance collaboration, inspire loyalty to an organization or an institution, and improve fairness.^16^^,^^33^^,^^38–41^ When women are supported into leadership positions, the effects can be transformational for everyone.^16^ Overall, women leaders positively impact 6 major areas of practice, based on findings of a recent systematic review: (1) financial performance, risk, and stability, (2) innovation, (3) engagement with ethical initiatives, (4) health, (5) organizational culture and climate outcomes, and (6) influence on other women’s careers and aspirations.^6^

Furthermore, hiring women leaders seems to leave a permanent imprint on the organizations that employ them, impacting both language used and local culture. This legacy helps dispel myths and challenge stereotypes, supporting more equity in the short-, medium-, and longer-term.^42^ Companies with a strong presence of female leadership and representation at the board level have shown evidence of and an emphasis on more sustainable corporate practices, largely due to female leaders’ ability to listen, share, collaborate, and provide a holistic view of problems, as well as strong problem-solving skills for complex challenges.^43^^,^^44^^,^^45^

## Women rising

Despite the known challenges and because of their known positive attributes as leaders, women are slowly but steadily rising in political strongholds and in corporate, healthcare, and educational organizations. In medical imaging and oncology increasing women representation is part of a growing agenda for fairer, more inclusive leadership.^46^^–^^50^ Many women have recently held or hold top positions of different professional bodies such as: (1) the Institute of Physics and Engineering in Medicine (IPEM), (2) the Royal College of Radiologists (RCR), (3) the Society and College of Radiographers (SCoR), (4) the British Institute of Radiology (BIR), (5) the European Congress of Radiology (ECR), (6) the European Federation of Radiographer Societies (EFRS), and (7) more related organizations and learned societies in North America and globally.^46–50^ For some of these organizations, it has been the first time a woman has taken the lead; others, however, have experienced longstanding, robust female leadership since their establishment. Overall, the trend shows increasing female representation in medical imaging and oncology settings. Their impact on the medical imaging and oncology organizations they lead extends to (1) novel and more accessible and inclusive education and practice,^46^ (2) focus on communication,^47^ person and patient care and members’ wellbeing,^48^ (3) emphasis on ethics, integrity and sustainability of clinical practice,^49^ (4) collaboration in scholarly research.^50^ There is an urgent need to harness the benefits that female leadership brings into healthcare, in general, and medical imaging and oncology, in particular, as sectors traditionally requiring caring and considerate leaders.^32^^,^^51^ Some ideas applicable to healthcare and other fields, are discussed below.

## How to help support more women in leadership roles

Different methods have been proposed to bring women into leadership roles and to help them sustain these positions for the longer-term. These include:

1. early talent recognition and nurturing, to ensure we develop and support them in the initial stages of their careers to maximize their impact and potential
2. establishing formal mentorship, sponsorship and allyship programmes and networks, to ensure engagement of the right people around each female mentee, so they can benefit from their experiences and guidance throughout their careers
3. funding in the form of leadership fellowships, training grants, research enabling grants, travel scholarships, and funding to support their entrepreneurial efforts
4. inclusive and ambitious educational provisions including continuing professional development for life
5. proportionate amendments of institutional policies and roles to ensure accessibility for and support of female leaders now and in the future
6. quality childcare provisions, equitable parental leave, flexible working patterns, and phased transition to work for those with caring commitments
7. social media campaigns of female role models^29^^,^^52–57^

Male allyship is also vital, as it can scale up impact quickly and extends beyond mentorship; it is about intentional actions to advance women in leadership positions that can start by individuals but need to progressively be incorporated into departmental and institutional values to end the gender gap. This might include making time to listen, creating clear pathways for women to rise, giving credit when it is due, calling out microaggressions and hidden bias, and creating female-friendly workspaces and work schedules that acknowledge female anatomy and physiology. For example, this involves understanding challenges related to room and equipment design, acknowledging the impact and toll of endometriosis, assisted reproduction, breastfeeding, and menopause on women’s health and wellbeing, and working with them to co-design and provide necessary adaptations. It also means organizing inclusive networking and team-building events that enable women to meet sponsors to advance their careers, advocating for inclusive conference facilities, and role-modelling equality in their own capacity.^29^^,^^52–64^

## Conclusion

Female leadership has evolved through years of exclusion and discrimination to a true act of caring for oneself, others and the society. There is a lot to be learned from other sectors and applied in healthcare contexts. Female leaders are improving individual and team culture in both communal traits, such as collaboration, equity, teamwork, inclusiveness, but also advance hard indicators of organizational performance, such as problem-solving, creativity, productivity, financial stability and sustainability, if they are given a chance. This chance needs to materialize into training and mentoring opportunities for accessing leadership positions, distinct career pathways and funding schemes to encourage female talent growth for life, and a strong network of allies, both male and female, to sustain these opportunities for the longer-term. It should also come with institutional changes to show true commitment into a fairer, more equitable and more inclusive future for everyone.
